# Assessment of Overall Survival Benefits in Patients Undergoing Complete Hepatectomy for Synchronous Colorectal Cancer With Liver and Lung Metastases

**DOI:** 10.6004/jadpro.2016.7.7.10

**Published:** 2016-11-01

**Authors:** Elsa Melissa Arvide, Jenilette Dames Velasco

**Affiliations:** The University of Texas MD Anderson Cancer Center, Houston, Texas

Overall survival (OS) is a statistical term referring to the percentage of a population in a group who are alive after a defined length of time, usually years. For example, a 5-year OS rate is the percentage of people who are alive 5 years after diagnosis or 5 years after the start of therapy. In surgical studies, OS is a universally accepted measure of direct benefit and can be easily and precisely measured. In general, OS is a common endpoint that is used to measure the success and efficacy of surgical treatments.

In recent decades, surgical treatment of stage IV colorectal cancer has evolved considerably, allowing patients to have longer OS (Zisis et al., 2013). Furthermore, in patients undergoing hepatic resection, improvements in systemic therapy for stage IV colorectal cancer have significantly doubled the OS rate (Kim et al., 2012). Hepatectomy is now performed in 25% of patients with colorectal liver metastases (CLM), resulting in a 5-year OS rate up to 58% (Abdalla et al., 2004; Choti et al., 2002; Kopetz et al., 2009; Pawlik et al., 2005). Unfortunately, the remaining 75% of patients with CLM will not undergo hepatectomy, for reasons such as the presence of extrahepatic metastases, most commonly lung metastases (Hecht et al., 2009).

The study by Mise et al. (2015a) discussed in depth in the article by Horner and Lencioni on page 781 if this issue addressed the benefits of resection of CLM in patients with synchronous lung metastases, even when the lung metastases are not resected. The findings of this study suggest the possibility of expanding the integration of hepatectomy and modifying the definition of resectability of CLM patients with synchronous colorectal liver and lung metastases.

## SUMMARY OF METHODS

In the Mise study, the main statistical approach includes Kaplan-Meier (K-M) analysis and Cox proportional hazard model analysis. This was the best approach for the analysis of the data because the K-M analysis provides an estimate survival curve of the different cohorts, which ultimately allow for interpretation of potential treatment planning when counseling patients. Additionally, the Cox proportional hazard model can further identify specific variables that would account for the differences we see in the survival curves.

In brief, the Cox analysis is a multivariate approach, and the K-M analysis is a univariate approach (Dudley, Wickham, & Coombs, 2016). In the Mise study, K-M analysis was used to calculate 3-year and 5-year OS from three main groups: CLM resection only, chemotherapy only, and resection of both liver and lung metastases.

The OS was calculated from the date of diagnosis of liver and lung metastases in patients with chemotherapy alone, the date of the hepatectomy for the study group (CLM resection only), and the date of hepatectomy and lung resection for patients who received combined resections. The OS differences between the three groups were compared using the log-rank test (Figure). This test is used to compare the survival distributions for the entire group based on the conventions on survival probabilities and censoring as the K-M analysis (Dudley et al., 2016). Unfortunately, the log-rank test only analyzes the significance between curves and does not provide an estimate of the size difference between groups, nor does it explore the effects of other clinical factors.

**Figure:**
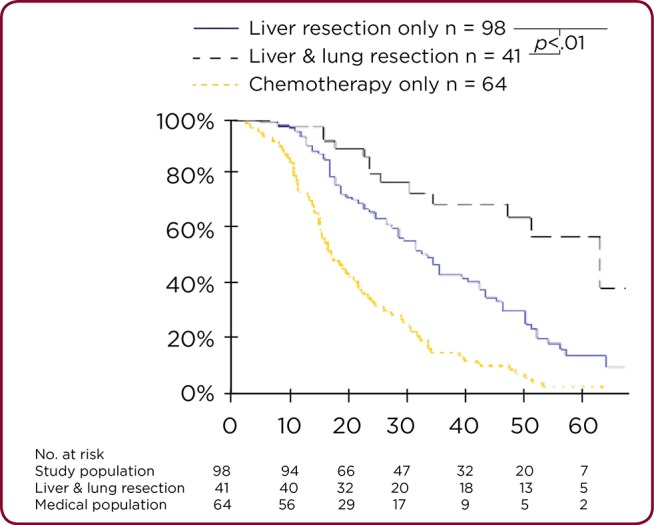
Overall survival of patients with synchronous colorectal liver and lung metastases who underwent resection of liver metastases only, stratified by the number of risk factors.

The clinicopathologic variables were evaluated in a univariate proportional hazard model to identify prognostic factors in the study population. All variables associated with survival with *p* < .01 within this univariate analysis were then entered into a Cox multivariate regression model with backward elimination. The Cox multivariate regression model is another strategy to analyze the effect of several independent variables over time. The multivariate analysis of patients in the CLM resection–only group revealed that *KRAS* mutation (hazard ratio [HR], 2.10; 95% confidence interval [CI] = 1.21–3.64; *p* < .01) and rectal primary tumor (HR, 1.72; 95% CI = 1.02–2.88; *p* = .04) were independent predictors of shorter OS. The survival of patients without these risk factors was similar to that of patients who underwent curative surgical resection.

## SUMMARY OF STUDY FINDINGS

In all, the 3-year and 5-year OS rates of patients with CLM resection were 42.9% and 13.1%, respectively, which was better than those for the patients treated with chemotherapy only (14.1% and 1.6%; *p* < .01). However, the OS rate of the CLM resection–only group was suboptimal when compared with that of patients post resection of both liver and lung metastases (68.9% and 56.9%; *p* < .01; see Table). This study demonstrated that complete resection of CLM without resection of synchronous lung metastases was associated with an intermediate survival between that of patients treated with chemotherapy alone (palliative intent) and those undergoing lung and liver resection (curative intent).

**Table:**
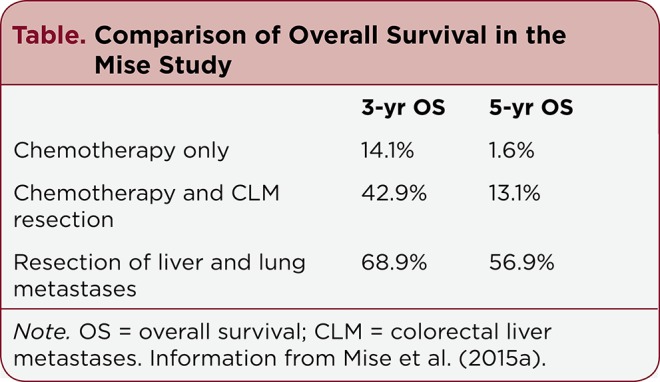
Comparison of Overall Survival in the Mise Study

**Study Strengths**

One of the strengths of the Mise study includes the fact that all patients were treated at the same cancer institution, therefore allowing consistency and uniformity in the standards and methodology of patient staging, imaging and laboratory studies, and interpretation of study findings. The Mise study is the first to report the outcome of CLM resection in the setting of unresected extrahepatic metastases. This study provides a surgical benchmark to which new surgical strategies can be compared and may potentially expand the possibility of hepatectomy in patients for whom surgical treatment was not previously indicated.

**Study Limitations**

A potential limitation of this study is the small study population (N = 98), which can undermine its overall strength. Having both a small sample size and a relatively short median follow-up duration (29 months) may provide a less accurate model of OS. The conclusion of this paper revealed that the combined liver and lung resection population had a greater OS than did the CLM-only group, and the OS of the CLM-only population was greater than that of the chemotherapy-only group.

The study subjects who underwent surgical treatments were chosen from a 12-year period, whereas the chemotherapy patients with unresectable CLM were chosen from a prospectively collected database, with no time frame indicated. This uncertain time period represents a significant limitation, considering the evolutions and variability in diagnosis and treatment of CLM over the past decade (Omura, 2008).

Additionally, the selection process of patients treated surgically and medically might have generated a more favorable prognosis. In comparison to the medical population, the study population included patients with fewer liver metastases, smaller lung metastases, and a lower preoperative carcinoembryonic antigen (CEA) tumor marker. Also, the indications for lung resection and the factors that predict OS were not consistent.

Within the CLM-only group, 45% had a *KRAS* mutation; however, the *KRAS* mutational status was not available for the other two comparison groups. Ongoing research continues to indicate that *KRAS* mutational status plays a pivotal role in tumor biology and disease progression (Kim et al., 2012). In fact, a recent study showed that *RAS* mutations predict radiologic and pathologic response in patients with chemotherapy before resection of CLM (Mise et al., 2015b). A retrospective *KRAS* mutational analysis on the patients in the chemotherapy-only group would aid in further supporting the results of the Mise study (2015a). One study demonstrated that patients with colorectal cancer who have a *KRAS* mutation had a shorter time to lung metastasis (Pereira et al., 2015). This supporting statement could potentially account for the decreased number of patients in that arm of the Mise study (2015a).

The Mise study alludes that KRAS-mutated tumors have a tendency to metastasize to the lungs and that the rates of *KRAS* mutations are higher in patients with lung metastases (De Roock et al., 2010; Vauthey et al., 2013). Furthermore, *KRAS* mutational status may not be the only contributing factor for predicting poor tumor biology. Ongoing studies are showing that the type of *KRAS* mutation (i.e., mutations in codon 13) can be associated with a decrease in the disease-free interval and OS (Dobre et al., 2015; Brudvik et al., 2015). Therefore, future studies and assessments must continue to regard the significant impact of tumor biology in treatment planning.

## PRACTICAL IMPLICATIONS

Although the conclusions drawn from this study are thought provoking, it is important to keep in mind that "OS" does not entirely determine patient prognosis. In the Mise study, OS only takes into account whether that patient presented to the clinic for follow-up (median follow-up, 29 months). It does not, however, give us further insight into the current status of the patient (i.e., alive with disease) or disease progression in the interim, both of which are important factors for patient counseling and education.

For surgical patients, OS statistics can only predict an estimated time frame of survival, but it does not include the quality of life after the procedure, nor does it indicate disease-free or progression-free survival. Due to the limitations of OS, additional endpoint measures such as disease-free and progression-free survival are often included in the outcome of studies.

Furthermore, this study points toward a growing trend: the importance of classifying tumor biology. Apart from a primary rectal tumor, this study demonstrates that *KRAS* mutational status is a significant prognostic factor with a worse OS. The KRAS protein is involved in regulating cell division and is widely recognized as a pro-oncogene (Genetic Home Reference, 2016). A *KRAS* mutational status in colorectal cancer has been widely studied and recognized as a poor prognostic factor (Shindoh et al., 2016). This study further confirms that tumor biology such as protein mutational status plays a significant and contributing role to predict OS and optimize patient selection for surgical treatments.

## CONCLUSION

Complete resection of metastases remains the primary goal of treatment for stage IV colorectal cancer. The current study by Mise et al. (2015a) suggests that patients with stage IV colorectal cancer and synchronous liver and lung metastases may have an additional treatment option, which could prolong OS. According to the study, patients undergoing complete resection of CLM without resection of synchronous lung metastases had a better OS than did patients treated with chemotherapy alone but worse survival than did patients undergoing complete metastasectomy. Furthermore, *KRAS* mutation status can potentially be used as a tool to predict and optimize patient selection for complete resection of CLM in the setting of synchronous lung metastases.

With the recent emergence of novel therapies for patients with advanced stage IV colorectal cancer, along with the increased utilization of molecular biomarkers, there remains a need for an individualized, multidisciplinary approach to handle the complex decision-making process for each patient. Both physicians and advanced practitioners play a significant role in treatment planning and patient education. Advanced practitioners provide quality patient care by offering detailed explanations of complex disease processes, available treatment options, prognostic factors, and ongoing research. This strategy ultimately helps each patient understand the disease process of their diagnosis as well as the reasoning behind the treatment plan. With the proper resources, patients can better understand and make more informed decisions regarding treatment choices, which will improve their OS and quality of life.
